# Diversity of helminths parasitizing *Phalacrocorax brasilianus* (Gmelin, 1789) in the Brazilian Amazon

**DOI:** 10.1590/S1984-29612024057

**Published:** 2024-09-23

**Authors:** Elaine Lopes de Carvalho, Ricardo Luis Sousa Santana, Tiago Paixão Mangas, Elane Guerreiro Giese

**Affiliations:** 1 Laboratório de Histologia e Embriologia Animal, Instituto da Saúde e Produção Animal, Universidade Federal Rural da Amazônia – UFRA, Belém, PA, Brasil; 2 Programa de Pós-graduação em Saúde e Produção Animal na Amazônia, Instituto da Saúde e Produção Animal, Universidade Federal Rural da Amazônia – UFRA, Belém, PA, Brasil; 3 Instituto Federal do Pará – IFPA, Breves, PA, Brasil

**Keywords:** Helminths, Phalacrocoracidae, Brazilian Amazon, Helmintos, Phalacrocoracidae, Amazônia brasileira

## Abstract

This study was carried out in northern Brazil to determine the prevalence of helminth parasites that infect *Phalacrocorax brasilianus* (Gmelin, 1789). Between July 2020 and July 2023, adult and larvae parasites were collected from the respiratory and gastrointestinal tract of 30 birds that died in fishing nets and in fishing corral in the municipality of Soure on Marajó Island. The identified parasites included the nematodes *Contracaecum* sp., *Contracaecum australe*, *Contracaecum rudolphii* sensu lato, *Contracaecum microcephalum*, *Contracaecum multipapillatum*, *Syncuaria squamata*, *Desportesius invaginatus*, *Tetrameres* sp., *Aplectana* sp., *Cyathostoma* sp., *Eucoleus contortus*, *Baruscapillaria spiculata*, *Baruscapillaria appendiculata*; the trematodes *Drepanocephalus spathans*, *Austrodiplostomum mordax*, *Austrodiplostomum compactum*, *Hysteromorpha triloba*; the cestodes *Paradilepis caballeroi*; and the acanthocephalans *Andracantha* sp., *Southwellina hispida* and *Southwellina macracanthus*. The whole prevalence was 96.66% (29/30) and the most frequent helminths were nematodes (96.66%; 29/30), followed by acanthocephalans (66.66%; 20/30). These data increase the knowledge about helminths in cormorants widely distributed to Marajó Island.

## Resumo

Este estudo foi realizado no norte do Brasil, para determinar a prevalência de helminto parasitas que infectam *Phalacrocorax brasilianus* (Gmelin, 1789). Entre julho de 2020 a julho de 2023, parasitos adultos e larvas foram coletados do trato respiratório e gastrointestinal de 30 espécimes dessas aves que morreram em redes de pesca e no curral de pesca, no município de Soure na Ilha de Marajó. Os parasitas identificados incluem os nematódeos *Contracaecum* sp., *Contracaecum australe*, *Contracaecum rudolphii sensu lato*, *Contracaecum microcephalum*, *Contracaecum multipapillatum*, *Syncuaria squamata*, *Desportesius invaginatus*, *Tetrameres* sp., *Aplectana* sp., *Cyathostoma* sp., *Eucoleus contortus*, *Baruscapillaria spiculata*, *Baruscapillaria appendiculata*; os trematódeos *Drepanocephalus spathans*, *Austrodiplostomum mordax*, *Austrodiplostomum compactum*, *Hysteromorpha triloba*; os cestódeos *Paradilepis caballeroi*; e os acantocéfalos *Andracantha* sp., *Southwellina hispida* e *Southwellina macracanthus*. O nível geral de infecção foi de 96,66% (29/30) e os helmintos mais presentes foram os nematódeos (96,66%; 29/30), seguidos dos Acantocéfalos (66,66%; 20/30). Esses dados aumentam o conhecimento sobre helmintos em cormorões amplamente distribuídos pela ilha de Marajó.

The order Suliformes includes four families; Fregatidae Degland & Gerbe, 1867, Sulidae Reichenbach, 1849, Anhingidae Reichenbach, 1849 and Phalacrocoracidae Reichenbach, 1849, with the latter distributed in southern USA and throughout South America (Monteiro, 2006; IUCN, 2016). *Phalacrocorax brasilianus* (Gmelin, 1789) (Syn. *Nannopterum brasilianus*), Neotropic cormorant, or locally known as “biguá”, inhabits inland waters and along the entire coast, rivers, lakes, estuaries, and mangroves, but does not move far from the coast (Souza et al., 2008).

The study developed by Monteiro et al. (2011) in Brazil, revealed a high diversity of species of parasites in these birds, recording 20 parasitic species, which has arised due to the wide geographic distribution of the genus *Phalacrocorax* Brisson, 1760. According to Monteiro (2006), the extremely rich helminth fauna in this species reflects a complex environment, rich in invertebrates and vertebrates that act as intermediate hosts in the life cycles of different species of parasites. *Phalacrocorax brasilianus* (Gmelin, 1789) are important agents of parasite dissemination due to their wide distribution (Sick, 1997). However, in Brazil, the helminth fauna of this cormorant still needs to be well defined.

The increasing number of cormorants within a territory are a possible reason for epizootics caused by parasites, generating the possibility of dissemination of these parasites to other hosts (Yakovleva et al., 2020).

As they are sentinel animals, marine birds are of great importance for ecological and epidemiological studies. Furthermore, parasites are strategic components of ecosystems and play important roles as they are part of the evolutionary cycle of many species of terrestrial and marine invertebrates and vertebrates (Matos et al., 2020).

This research aimed to record the occurrence of parasitic helminths in *P. brasilianus*, contributing with data on the parasitic biodiversity of this bird in Northern Brazil.

**Figure 1:**
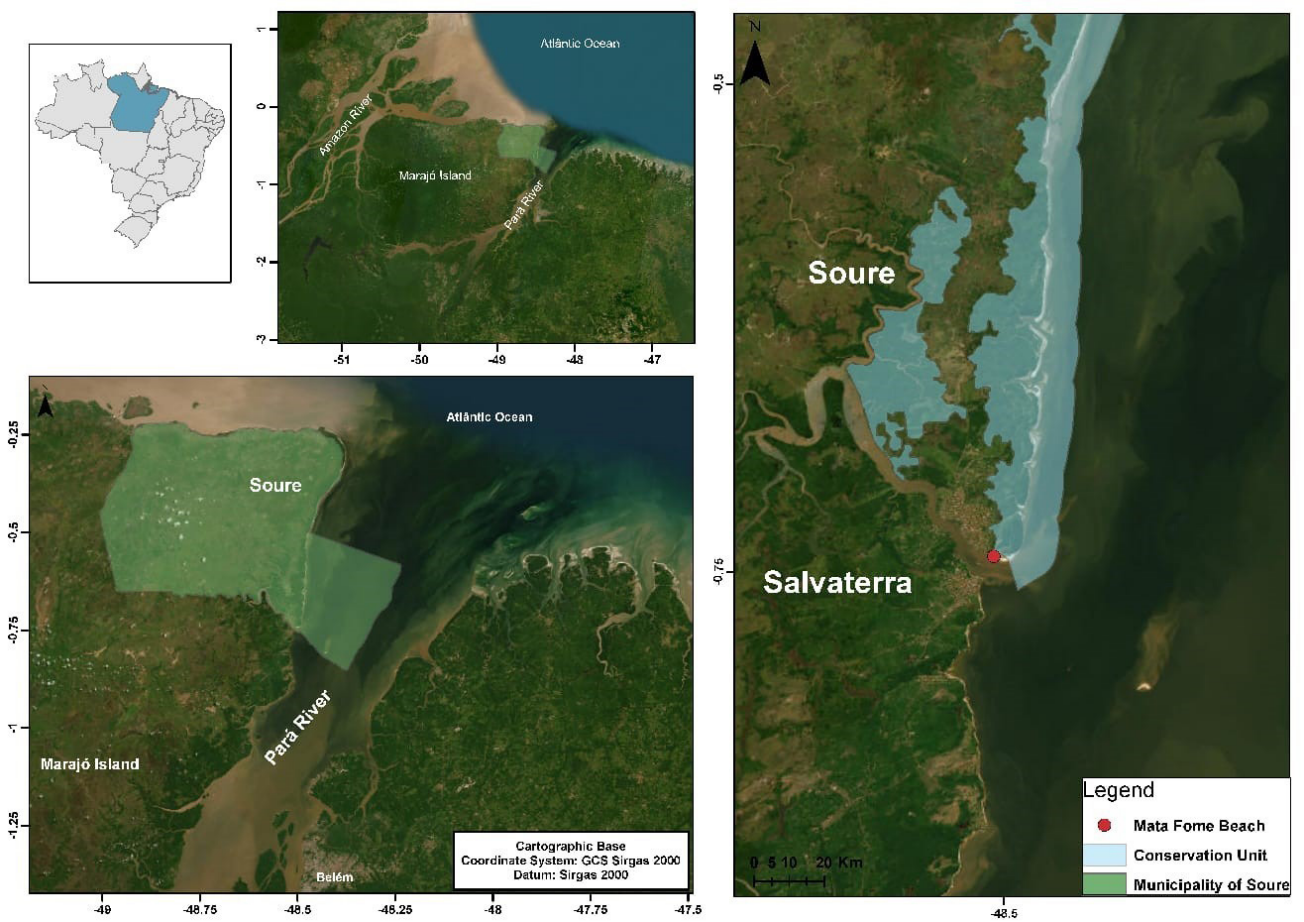
Geographic location of Marajó Island with identification of the Marine Extractive Reserve in the municipality of Soure, Marajó Island, State of Pará.

Thirty specimens (19 males and 11 females) of *P. brasilianus* were acquired from the Marine Extractive Reserve in the Municipality of Soure (Figure 1). The birds were found trapped in fishing nets or pens owned by some members of the fishermen's association in the Municipality of Soure, State of Pará, Brazil. Only the respiratory and digestive tract organs were disposed under refrigeration and sent to the laboratory for dissection. In the laboratory, the organs were separated and placed in Petri dishes with 0.9% NaCl saline solution and examined individually using a stereomicroscope (Leica ES2).

The collected nematodes were washed in distilled water, fixed and stored in A.F.A. solution (93 parts of 70% ethanol, 5 parts of 37% formaldehyde and 2 parts of glacial acetic acid). For light microscopy, nematodes were clarified in 0.5% Amann’s Lactophenol solution and observed under a Leica DM2500 microscope with a drawing tube, after which they were stored in ethanol-glycerin (50 parts of 70% ethanol and 50 parts of pure glycerin). Live nematodes were fixed in warm A.F.A. (~65 °C) to avoid contraction and facilitate morphological analysis.

Live acanthocephalans were kept in distilled water for 24 hours in the refrigerator to allow them to evert proboscis (Amato & Amato, 2010). Acanthocephalans and medium-sized trematodes were compressed between slide and coverslip in a Petri dish with A.F.A. fixative. The compression period was evaluated according to the thickness of the helminth (Oyarzún-Ruiz & González-Acuña, 2020). The live cestodes were placed in distilled water and taken to the refrigerator (for 6 hours) to die with their muscles relaxed, then fixed in A.F.A. without compression (Amato & Amato, 2010).

Platyhelminthes and acanthocephalans were stained in alcoholic carmine and Gomori trichrome, respectively, according to Amato & Amato (2010) and Oyarzún-Ruiz & González-Acuña (2020), all worms were mounted individually between a slide and coverslip with the mounting medium Erv-Mount^®^. Acanthocephalans were clarified with Amann's Lactophenol and temporarily mounted between the slide and coverslip.

Taxonomic studies of nematodes, trematodes, cestodes and acanthocephalans were in accordance with Yamaguti (1963, 1971), Khalil et al. (1994), Gibson et al. (2002) and Anderson et al. (2009).

Photomicrographs were obtained using a LEICA DM2500 microscope with an attached LEICA type DFC310 FX camera. The boards made up of drawings and images obtained with the photomicroscope were used using Adobe Photoshop CS^®^ software.

To determine the ecological indices of parasitism, prevalence (%), mean intensity of infection (_M_I) and mean abundance (_M_A) were estimated according to Bush et al. (1997).

Between June 2020 to July 2023, the birds were collected under authorization for activities with scientific purposes from ICMBio/SISBIO nº 74195, and in accordance with the Ethics Committee on the Use of Animals under protocol nº 6309230520 of the Universidade Federal Rural da Amazônia.

**Figure 2:**
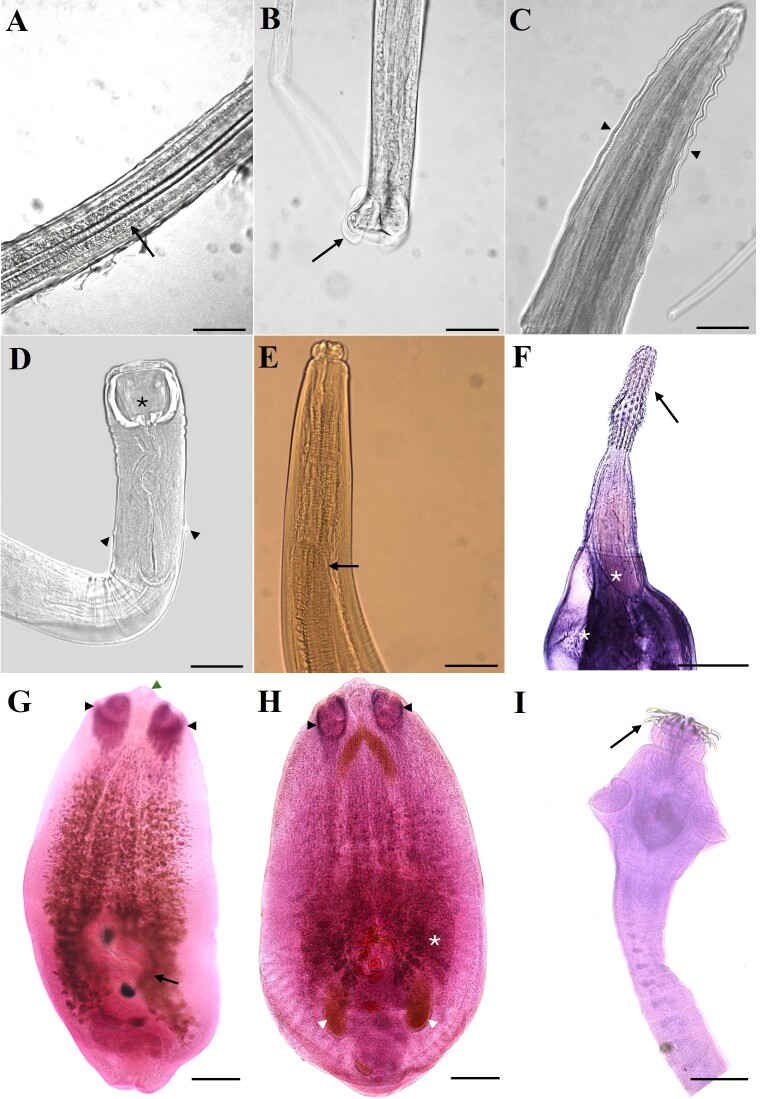
Helminths in *Phalacrocorax brasilianus*. (A) *Baruscapillaria spiculata*, stellate spicular sheath (arrow). Scale bar: 50 µm; (B) *Baruscapillaria appendiculata*, posterior region, tail of the male (arrow). Scale bar: 50 µm; (C) *Syncuaria squamata*, cephalic cords (arrowhead). Scale bar: 100 µm; (D) *Cyathostoma* sp., buccal capsule (*) and deirids (arrowhead). Scale bar: 200 µm; (E) *Contracaecum* sp., intestinal cecum (arrow). Scale bar: 200 µm; (F) *Andracantha* sp., proboscis (arrow) and necklace of spines (*). Scale bar: 200 µm; (G) *Austrodiplostomum mordax*, two pseudo suckers (arrowhead) well developed, oral sucker (arrowhead green) and ventral sucker (arrow). Scale bar: 200 µm; (H) *Austrodiplostomum compactum*, two pseudo suckers well developed (arrowhead), ventral sucker (*) and intestinal cecum (arrowhead white). Scale bar: 200 µm; (I) *Paradilepis caballeroi*, scolex with hooks on the rostellum (arrow). Scale bar: 200 µm.

Of the 30 cormorants analyzed, 36.67% (11/30) belonged to females and 63.33% (19/30) to males. Of these, 96.66% (29/30) of birds were found parasitized by helminths. The Phylum Nematoda was the most representative group, occurring in all the parasitized birds; followed by Phylum Acantocephala, Class Cestoda and Class Trematoda with 66.66% (20/30), 23.33% (7/30) and 16.66% (5/30) of the analyzed birds, respectively (Table 1, Figure 2).

**Table 1:** Location, prevalence, average abundance and average intensity of parasites found in *Phalacrocorax brasilianus* (n=30) obtained in the municipality of Soure, Marajó Island, State of Pará.

Taxa/Parasite	Site of infection	Prevalence %	Medium abundance	Medium intensity	Amplitude
*Baruscapillaria appendiculata*Freitas, 1933; Moravec, Salgado Maldonado & Osorio Sarabia, 2000	cloaca	93.3	11	11.8	7-84
*Baruscapillaria spiculata* (Freitas, 1933) Moravec 1982	intestine, cloaca	23.3	1.1	4.7	1-9
*Eucoleus contortus* (Creplin, 1839)	esophagus	6.7	0.6	8.5	4-13
*Tetrameres* sp.	proventriculus	13.3	1.8	13.8	7-21
*Contracaecum* sp.	proventriculus, ventriculus	26.7	10.9	41.1	22-65
*Contracaecum microcephalum* (Rudolphi, 1809) Baylis, 1920		3.3	0.3	9	9
*Contracaecum rudolphii* Hartwich, 1964 sensu lato		56.7	7.7	13.6	3-29
*Contracaecum multipapillatum* (Drasche, 1882) Lucker, 1941		6.7	0.1	1.5	1-2
*Contracaecum australe* Garbin, Mattiucci, Paoletti, González-Acuña & Nascetti, 2011		83.3	35.1	42.1	7-360
*Syncuaria squamata* von Linstow, 1883	ventriculus	20	0.5	2.3	1-4
*Desportesius invaginatus* (von Linstow, 1901)		6.7	0.07	1	1
*Aplectana* sp.	ventriculus	3.3	0.2	5	5
*Cyathostoma* sp.	trachea	36.7	0.7	1.9	1-3
*Austrodiplostomum mordax* Szidat & Nani, 1951	intestine	6.7	0.6	9	5-13
*Austrodiplostomum compactum* (Lutz, 1928) Dubois, 1970	intestine	6.7	0.8	11.5	9-14
*Hysteromorpha triloba* (Rudolphi, 1819) Lutz, 1931	intestine	3.3	0.07	1	2
*Drepanocephalus spathans* Dietz, 1909	intestine	6.7	0.3	4	3-5
Family Gorgoderidae	intestine	3.3	0.03	1	1
*Paradilepis caballeroi* Rysavy & Macko, 1971	intestine	26.7	2.9	10.9	1-30
*Andracantha* sp.	small intestine, large intestine	36.7	7.9	21.5	2-37
*Southwellina hispida* (Van Cleave, 1925)	small intestine	30	3.9	13	5-23
*Southwellina macracanthus* (Ward & Winter, 1952)	intestine, cecum	10	2.7	26.7	10-47

Monteiro et al. (2011) found a helminth fauna with 21 taxa, so our study on Marajó Island were in accordance with these authors, although that study was carried out in different regions of Brazil. Overall, these results indicate that the communities were similar in structure at the component level, which is attributed to cormorants feeding on similar fish species. Differences at the level of the infracommunity can be attributed to differences in the degree of dominance, as the richest and most diverse infracommunities were those that showed greater uniformity in the abundance of species (Violante-González et al., 2011).

Of the nematodes recorded, *Baruscapillaria appendiculata* (Freitas, 1933) Moravec, 1982 and *B. spiculata* (Freitas, 1933) Moravec, 1982 were found previously in Rio de Janeiro, in the intestine of *P. brasilianus* by Freitas (1933). Garbin et al. (2021) redescribed the taxon *B. spiculata* in cormorants from Argentina.

The genus *Aplectana* Railliet & Henry 1916 comprises 60 species (Sou & Sow, 2019; Chen et al., 2021; Santos et al., 2023) of which 27 are known from Central and South America. These nematodes are intestinal parasites of amphibians and reptiles (Falcón-Ordaz et al., 2014). In our research we recorded the occurrence of this genus in birds, probably due to the bird's diet including amphibians.

In Brazil, adult specimens of *Austrodiplostomum compactum* (Lutz, 1928) Dubois, 1970 were found in *P. brasilianus* by Monteiro et al. (2011) from the “Cerrado” biome. In addition to *A. compactum*, *A. mordax* Szidat & Nani, 1951, *Drepanocephalus spathans* Dietz, 1909 and *Hysteromorpha triloba* (Rudolphi, 1819) Lutz, 1931, and a member of the Gorgoderidae family without specific identification, similar to Monteiro (2006). Family Gorgoderidae is relatively large and infects a wide range of freshwater and marine vertebrates as definitive hosts, including amphibians, chondrichthids, teleosts, and reptiles (Campbell, 2008). The adult digenetic parasites recorded in these birds, and the metacercariae larvae, have fish as paratenic hosts according to Monteiro (2006). Another parasite Platyhelminthes recorded in our research was the cestode *Paradilepis caballeroi* Rysavy & Macko, 1971 also isolated by Monteiro et al. (2011) and González-Acuña et al. (2020).

Studies of the parasitic fauna of the neotropical cormorant in South America have been carried out mainly in Brazil (Monteiro et al., 2011) and Argentina (Drago et al., 2011). Besides, González-Acuña et al. (2020) identified 12 species of parasites in Chile, including *H. triloba*, *Andracantha* sp., *Cyathostoma phenisci* Baudet, 1937 and *P. caballeroi*, records that are similar to our findings.

In this research, the helminthological fauna of *P. brasilianus* include taxa of helminths which could be considered typical of genus *Phalacrocorax* such as the nematodes *Eucoleus contortus* (Creplin, 1839), *Baruscapillaria* spp., *Tetrameres* sp., *Contracaecum rudolphii* Hartwich, 1964, *Syncuaria squamata* von Linstow, 1883 and the acanthocephalans of genus *Andracantha*.

For the first time, the taxa *Aplectana* sp. (accidental taxon), *Desportesius invaginatus* (von Linstow, 1901), *Contracaecum microcephalum* (Rudolphi, 1809) Baylis, 1920, *Contracaecum multipapillatum* (Drasche, 1882) Lucker, 1941, *Contracaecum australe* Garbin, Mattiucci, Paoletti, González-Acuña & Nascetti, 2011 and *Cyathostoma* sp. were recorded in *P. brasilianus* from Brazil.
